# Contraction of T cell richness in lung cancer brain metastases

**DOI:** 10.1038/s41598-018-20622-8

**Published:** 2018-02-01

**Authors:** Aaron S. Mansfield, Hongzheng Ren, Shari Sutor, Vivekananda Sarangi, Asha Nair, Jaime Davila, Laura R. Elsbernd, Julia B. Udell, Roxana S. Dronca, Sean Park, Svetomir N. Markovic, Zhifu Sun, Kevin C. Halling, Wendy K. Nevala, Marie Christine Aubry, Haidong Dong, Jin Jen

**Affiliations:** 1 Division of Medical Oncology, Mayo Clinic, Rochester, MN USA; 2 Department of Laboratory Medicine and Pathology, Mayo Clinic, Rochester, MN USA; 3 Department of Immunology, Mayo Clinic, Rochester, MN USA; 4 Department of Health Sciences Research, Mayo Clinic, Rochester, MN USA; 5 Center for International Blood and Marrow Transplant Research, Minneapolis, MN USA; 6 Department of Radiation Oncology, Mayo Clinic, Rochester, MN USA; 7 Genome Analysis Core and the Biomarker Discovery Program, Center for Individualized Medicine, Mayo Clinic, Rochester, MN USA

## Abstract

Very little is known about how the adaptive immune system responds to clonal evolution and tumor heterogeneity in non-small cell lung cancer. We profiled the T-cell receptor β complementarity determining region 3 in 20 patients with fully resected non-small cell lung cancer primary lesions and paired brain metastases. We characterized the richness, abundance and overlap of T cell clones between pairs, in addition to the tumor mutation burden and predicted neoantigens. We found a significant contraction in the number of unique T cell clones in brain metastases compared to paired primary cancers. The vast majority of T cell clones were specific to a single lesion, and there was minimal overlap in T cell clones between paired lesions. Despite the contraction in the number of T cell clones, brain metastases had higher non-synonymous mutation burdens than primary lesions. Our results suggest that there is greater richness of T cell clones in primary lung cancers than their paired metastases despite the higher mutation burden observed in metastatic lesions. These results may have implications for immunotherapy.

## Introduction

Advances in genomic profiling have facilitated the molecular characterization of tumor heterogeneity in many types of cancers. Although the implications of spatial and temporal tumor heterogeneity may not yet be fully understood, clonal evolution likely affects prognosis, treatment selection, therapeutic response and treatment resistance^1–3^. Despite our burgeoning understanding of tumor heterogeneity, very little is known about the dynamics of tumor immunogenicity and the repertoire of the adaptive immune response to metastatic non-small cell lung cancer (NSCLC).

The discovery of programmed cell death 1 ligand 1 (PD-L1)^4^ and its effects on T cell function and survival^5^ have revolutionized cancer therapeutics. There are three drugs that inhibit PD-L1 or its receptor PD-1 that are approved by the FDA for the treatment of metastatic NSCLC^6^ and many others agents are in development. PD-L1 expression by tumor cells has been explored as a predictive biomarker for patients to receive these agents, but there is significant confusion about the clinical applicability of discrepant PD-L1 expression between paired lesions^7^. Many issues including the dynamics and context of PD-L1 expression^8^, the size of a specimen^9^, the timing of specimen acquisition in relation to treatment, and the agreement between assays all contribute to this confusion^10,11^. Additionally, we have reported that PD-L1 expression can be temporally dynamic^12^ and is heterogeneous between multifocal lung cancers^13^ and between paired primary lesions and brain metastases^14^. During these studies we noticed that there was significant variability in tumor infiltration by lymphocytes between paired primary lesions and brain metastases. Accordingly, we sought to assess the distribution of T cell clones between paired NSCLC primary lesions and brain metastases in order to characterize the temporal and spatial relatedness of the adaptive immune response.

## Results

### Brain metastases have significantly fewer T cell clones than paired primary lesions

**Figure 1:**
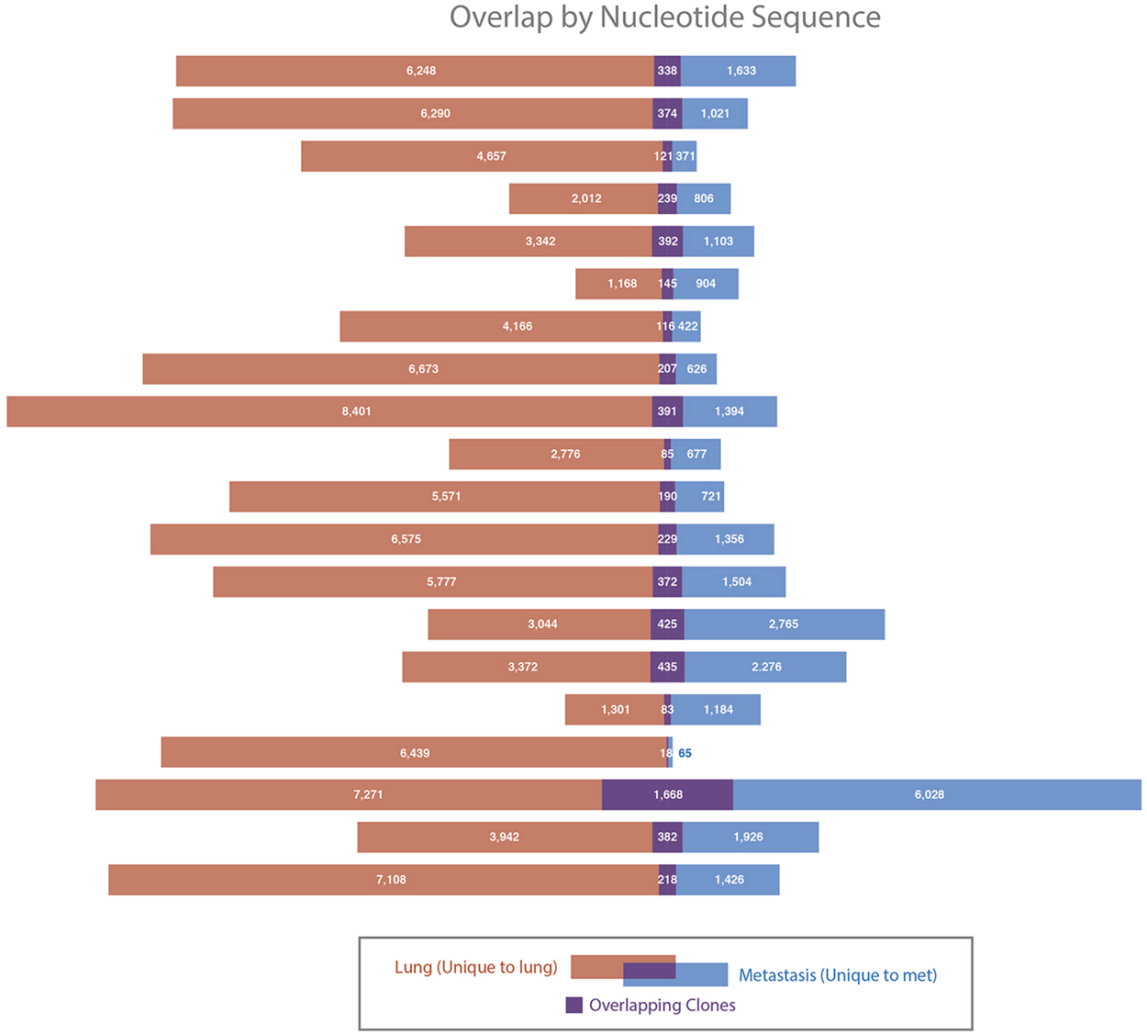
Comparative T cell richness and overlap. Each bar represents the detected T cell clones in one subject’s paired specimens. The unique T cell clones in the primary lesions are shown in red, the unique T cell clones in the brain metastasis are shown in blue, and the overlapping clones are in purple.

To evaluate the distribution of T cell clones between primary and metastatic sites, we identified a cohort of 20 patients with metastatic NSCLC who underwent surgical resection of their primary and metastatic lesions either because of presentation with synchronous oligometastatic disease, or delayed recurrence of a solitary brain metastasis (Table 1). There was a significant contraction in the number of unique, productive T cell clones in paired brain metastases (median 1540, range 83–7696) compared to primary lesions (median 4551, range 1049–8939; mean of differences −2803, 95% CI −4202 to −1405; p = 0.0005; Fig. 1). Similarly, fewer T cells detected by IHC were observed in brain metastases than primary lung cancers (mean of differences −12, SD 16; p = 0.003).

**Table 1:** Patient characteristics.

	N (%) or median and interquartile range
Age at first diagnosis	57.5 years (50.3–69.3)
Sex	
Male	9 (45%)
Female	11 (55%)
Histology	
Adenocarcinoma	20 (100%)
Squamous cell carcinoma	0 (0%)
Interval between primary and metastasis specimen acquisition	348 days (146–910)
History of Tobacco Use	
Yes	17 (85%)
No	3 (15%)
Pack-Years	40 (26–58)
Characteristics for the subset of 13 patients with TMB and NeoAg data	
Age at first diagnosis	57 years (49–63)
Sex	
Male	6 (46%)
Female	7 (54%)
Histology	
Adenocarcinoma	13 (100%)
Squamous cell carcinoma	0 (0%)
Interval between primary and metastasis specimen acquisition	401 days (155–1015)
History of Tobacco Use	
Yes	10 (77)
No	3 (23)
Pack-Years	30 (12.5–65)

There was a moderate correlation between T cells detected by IHC for CD3 and the total number of productive clones amongst all specimens (Spearman ρ = 0.45, p = 0.004).

Since our analysis of multiple 10 micron sections of each lesion may not encompass all of the T cell clones within a tumor, we used iChao1 and the Efron-Thisted Estimator to estimate the total number of unique productive T cell clones in each lesion. Both iChao1 (mean of differences −20,355, 95% CI −29,561 to −11,149; p = 0.0002) and the Efron-Thisted Estimator (mean of differences −14,273, 95% CI −21331 to −7216; p = 0.0004) estimated that there is a significant decrease in the number of unique productive T cell clones in metastatic lesions.

### Dominant T cell clones are more abundant in brain metastases than paired primary lesions

**Figure 2:**
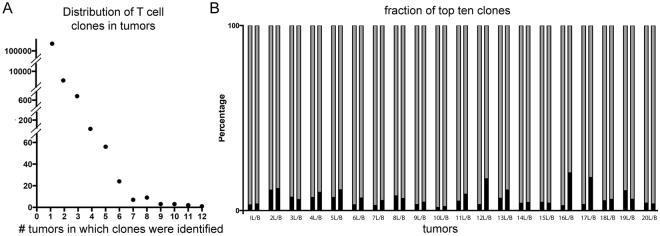
Clonal distributions. Unique T cell clones are plotted by the number of tumors (x axis) in which they were identified. (**A**) The vast majority of clones were only observed in one tumor. The relative fraction of the top ten clones (black bars) is plotted against the total number of T cells (gray bars) in matched pairs of lung tumors [L] and brain metastases [B] for all 20 cases (**B**).

To evaluate the distribution of T cell clones within each lesion, we assessed the evenness of clonal abundance and scored each lesion with heterogeneity indices. The vast majority of T cell clones when assessed by predicted unique amino acid sequences were specific to a single lesion (108,165/117,736, 91.87%; Fig. 2A). Overall, there was no significant difference in Pielou’s Evenness (mean of differences −0.004778, 95% CI −0.02 to 0.01; p = 0.51) or Simpson’s Diversity Index (mean of differences 0.002, 95% CI 0.001 to 0.004; p = 0.05) between primary and metastatic pairs, but scores for these indices were very low overall. Since Pileou’s Evenness Index and Simpson’s Diversity Index are likely influenced by the very large number of unique clones in each specimen, we also compared the relative abundance of the ten most abundant T cell clones for each lesion between pairs. We observed that there is a significant increase in abundance of the ten most common T cell clones in metastases compared to primary lesions (mean of differences 3.19, 95% CI 0.50 to 5.88; p = 0.03; Fig. 2B). In other words, there is a greater degree of clonal expansion of the ten most abundant T cell clones in brain metastases than primary lung cancers. One pair of lesions shared two clones of their ten most abundant, and two pairs of lesions each shared one clone of their respective ten most abundant. With the exception of these four clones, no others were one of the ten most abundant clones in any lesion. In other words, the most abundant clones were almost always unique to a single lesion.

### Clonal overlap is limited between paired lesions

To evaluate the spatial heterogeneity of the adaptive immune response, paired primary and metastatic lesions were analyzed for the presence of the same T cell clones. Few T cell clones identified in primary lesions were also found in brain metastases, and vice versa (mean Morisita Index 0.23, 95% CI 0.15–0.31; Fig. 1). As stated above, the majority of the detected T cell clones were unique to the lesion in which they were detected (Fig. 2A).

### Tumor mutation burden is higher in paired brain metastases than primary lung cancers

**Figure 3:**
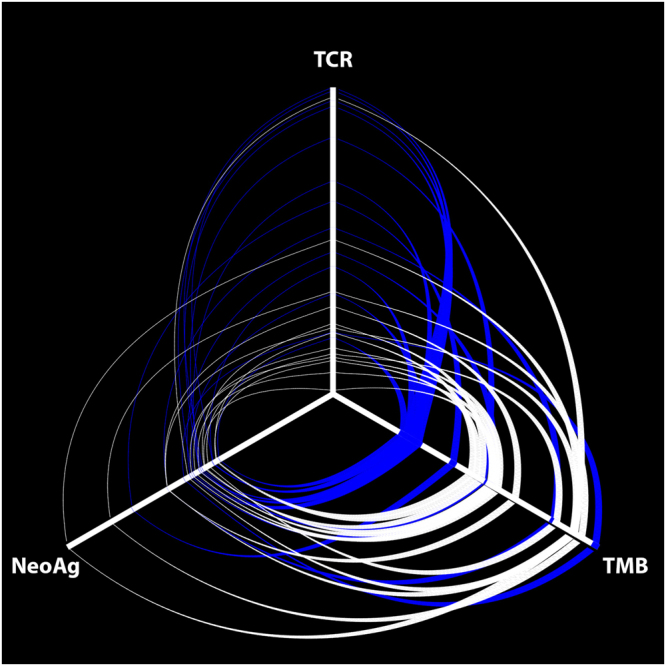
Hive plot of T cell clonality, tumor mutation burden and neoantigen prediction. The number of unique, productive T cell clones (TCR), the tumor mutation burden (TMB) and the predicted neoantigen load (NeoAg) for primary lung cancers (blue lines) and brain metastases (white lines) are plotted along each axis of this hive plot. The central point represents a value of zero for all axes. The axes have been normalized such that the ends represent the highest value for that measurement.

To determine whether the differences in distributions of T cell clones were associated with tumor mutation burden (TMB), we compared the non-synonymous TMB between 13 paired lesions with sufficient DNA for tumor sequencing. Overall, there was a significantly higher TMB in brain metastases (median 24.9/Mb, interquartile range [IQR] 23.0–36.6/Mb) than in paired primary lung cancers (median 12.5/Mb, IQR 11.3–23.2/Mb, p < 0.0001; Fig. 3). The concordance in mutations between pairs was high (median 85.7%, IQR 80.6–88.2%). Overall, there was no correlation between TMB and T cell richness in lung cancer primaries (Spearman ρ = −0.18, p = 0.55), but there was a correlation in brain metastases (Spearman ρ = 0.65, p = 0.018). These non-synonymous tumor mutations were used to predict potential tumor neoantigens for MHC class I alleles. Despite the higher TMB, brain metastases did not have a statistically significant higher predicted neoantigen load (median 898, IQR 825–1081) than paired primary lung cancers (median 874, IQR 743–953; p = 0.20).

## Discussion

We identified that there are fewer T cell clones in brain metastases than in paired primary lung cancers despite an increase in the non-synonymous mutational burden. Additionally, the distribution of T cell clones is markedly different between sites, with the ten most abundant clones representing a larger proportion of all T cells in metastases than primary lesions. Overall, few clones were shared between paired sites suggesting that there is some degree of T cell clonal expansion within a metastasis and divergent tumor immunogenicity associated with the metastatic process. It is not certain whether these findings are due to restricted entry of T cells through the blood brain barrier, the capabilities of microglial cells to present tumor antigens and migrate to lymph nodes, or other mechanisms of immune evasion; however, these differences may not be related to the distribution of potential neoantigens since brain metastases had a higher non-synonymous mutation burden but an equivalent predicted neoantigen load.

We initially hypothesized that the majority of the detected T cell clones in metastatic lesions would also be found in the paired primary lesions. The majority of clones we detected were unique to the lesion they were detected in, suggesting that there is significant immunogenic diversity between the lesions included in our cohort. Thus our data suggest that there is ongoing evolution of tumor clones at each site resulting in divergent tumor immunogenicity following metastasis. Even though we detected a higher non-synonymous TMB, we did not find a statistically significant higher predicted neoantigen load in brain metastases. This is possibly because we only determined neoantigens that may bind MHC class I alleles and not MHC class II alleles. At present neoantigen prediction is currently challenged by the high failure rate of these predictions^15^. Accordingly it remains unknown as to whether the method we used for neoantigen analysis is truly predictive of the neoantigen load. A recent study profiled the T cell repertoire in multiple regions of 11 localized adenocarcinomas of the lung^16^. The authors reported a similar number of unique TCRβ rearrangements per sample among the primary tumors compared to our study. In contrast to the reported positive correlation between T cell clones and tumor neoantigen heterogeneity within the primary lung cancers, we observed a contraction of the T cell repertoire in brain metastases despite a higher TMB and equivalent predicted neoantigen load. Although we agree with the conclusions of the other work that spatial differences in the T cell repertoire may be driven by distinct neoantigens in different tumor regions of primary tumors, our findings in brain metastases limit the scope of this conclusion.

To put our results into context, it is important to consider immunologic privilege and the blood brain barrier. An immune-privileged site is defined as one that does not reject implanted tissue grafts through an immune response^17^. The central nervous system has long been considered an immune-privileged site, but this view has become more nuanced and complex with the demonstration of activated circulating T cells that cross the blood-brain barrier, rejection of tissue grafts placed in cerebral ventricles, and drainage of cerebrospinal fluid into extracranial lymph nodes^18^. Models of experimental auto-immune encephalitis suggest that there is efficient antigen sampling within the central nervous system, but efferent immunity or leukocyte recruitment is restricted^19^. This model is consistent with our observation of fewer T cell clones in brain metastases than primary tumors. In a study of melanoma it was shown that tumors that are not infiltrated with T cells have similar frequencies of potentially immunogenic, nonsynonymous somatic mutations as tumors that are infiltrated with T cells^20^. Since the metastatic lesions in our cohort had a higher nonsynonymous somatic mutation burden compared to their paired primary lesions, it is possible that diminished antigen presentation or restricted efferent immunity reduced the accumulation of T cells in the metastatic lesions within the brain.

Lung cancer is the most frequent cause of brain metastases which are detected in about 15–20% of patients with this diagnosis^21,22^. Survival is very poor after the detection of brain metastases^23,24^, and treatment is complicated by the blood brain barrier. Experiments with primary brain cancers highlight the significance of the blood brain barrier. More specifically, orthotopic and heterotopic glioblastoma multiforme xenografts have differential responses to therapeutics such that heterotopic xenografts in murine flanks frequently respond to treatments even though orthotopic xenografts do not^25–27^. Regardless, given the potential of various immune cells to cross the blood brain barrier, adoptive cell therapies are in development for the treatment of glioblastoma multiforme^28^. Since the goal of many novel immunotherapeutics is to enhance an existing, adaptive, antitumor immune response, the contraction of T cell clonality in brain metastases may limit the applicability of these approaches. Regardless, there were responses in the brain metastases of six of 18 patients (32%) with NSCLC in a small open-label clinical trial with the PD-1 inhibitor pembrolizumab^29^. Interestingly, there are cases of mixed responses between primary and metastatic lesions^30^.

Although our study only included 40 specimens from 20 patients, we observed large differences in the richness and abundance of T cell clones and non-synonymous TMB. Additionally, 18 of the 20 patients did not receive interval therapies, so it is challenging to assess how systemic chemotherapy, whole brain radiotherapy or stereotactic radiotherapy may have affected T cell accumulation following one of these interventions. Similarly, it is difficult to control for the effect of corticosteroids that are commonly administered before neurosurgery. Furthermore, our study was limited only to patients with resected brain metastases. It would be instructive to determine whether there is a similar contraction of T cell clonality at other metastatic sites. Although we extracted DNA from different sites, the quality of DNA was similar between pairs. Similar to what we observed in a larger cohort of paired brain metastases and primary lung cancers^14^, there were fewer tumor infiltrating lymphocytes detected by IHC in the metastatic lesions than primary lesions. Also, the number of tumor-infiltrating lymphocytes identified by IHC generally correlated with the number of T cell clones. Our specimens were all formalin-fixed and paraffin-embedded (FFPE). Since others have noted that DNA fragmentation in FFPE samples limits recovery of T cells^31^, we may not have retrieved the complete infiltrating T cell repertoire.

Overall our results indicate that there is greater richness but less relative abundance of T cell clones in primary NSCLC lesions compared to paired brain metastases despite an increase in the TMB in metastatic lesions. Strategies to overcome the immunogenicity of brain metastases or improve trafficking of T cells to brain metastases may improve outcomes with immunotherapy.

## Methods

### Patient selection and pathology review

We identified 20 patients with NSCLC and paired, fully resected primary and metastatic tumors through review of available specimens within Mayo Clinic’s Tissue Registry which were used in a previous study^14^. These specimens were collected per institutional protocols, and use of these specimens was approved by Mayo Clinic’s Institutional Review Board (#13-007990). A pathologist (MCA) reviewed specimens for presence of tumor and tumor percentage. We excluded patients with a history of multiple malignancies in order to reduce the possibility of including cancers other than NSCLC. Patient characteristics are summarized in Table 1.

### DNA purification

DNA was isolated from FFPE tissue samples using the AllPrep® DNA/RNA FFPE Kit (Qiagen, Hilden, Germany). The hemotoxylin and eosin stained slides of each case were reviewed for tumor and non-malignant tissue and marked accordingly under the microscope. Five unstained tissue sections (10 µm thick) were deparaffinized in xylene and 100% ethanol (twice in each for 10 minutes). The macrodissected tumor areas of the deparaffinized tissues were placecd into a 1.5 ml collection tubes for DNA and RNA extractions following the manufacturer’s protocol. The DNA samples were quantified by Nano Drop 1000 Spectrophotometer (Thermo Scientific, Wilmington, DE, USA). The fragmentation sizes were evaluated by the Agilent 2200 Tape Station system using the Genomic DNA Screen Tape Assay (Agilent Technologies, Santa Clara, CA, USA). There was no significant difference in the DNA Integrity Number between primary lung cancers (mean 3.8, SD 1.0) and paired brain metastases (mean 3.8, SD 0.8; p = 0.92) and the ratio of absorbance at 260 nm to 280 nm was between 1.8 and 2.0 for all specimens (Supplemental Data).

### TCRβ amplification and sequencing

T cell receptor profiling was performed per protocol with ImmunoSEQ (Adaptive Biotechnologies, hsTCRβ Kit). Two sets of PCRs were performed using DNA extracted from the tumors following the manufacturer’s protocol (Adaptive Biotechnologies kit instructions and components). Based on availability, 2.24–4.92 µg gDNA were used in the initial PCR with paired samples having the same total DNA input. The initial PCR used a mix of multiplexed V- and J-gene primers which amplify all possible recombined receptor sequences from the DNA sample. This was followed by the second PCR amplification to incorporate the unique molecular barcodes to each PCR product. The samples were pooled together with a negative and a positive control and then sequenced on an Illumina MiSeq platform using a 100 cycles paired end protocol and sequence-ready primers provided by Adaptive Biotechnologies. After sequencing the raw data were transferred to Adaptive Biotechnologies and processed into a report that includes a normalized and annotated TCRβ profile repertoire (Supplemental Data).

Whole Exome Sequencing of primary lung tumors and matched brain metastases.

A total of 100ng purified genomic DNA from each sample was used for library construction using the NEB Ultra II Kit and then subjected to whole exome capture using Agilent All Exon v5 plus UTR kit following the manufacturer’s protocol. The resulting libraries were quantified and subjected to 100 cycles of paired end sequencing at three samples per lane on HiSeq2500.

Methods for DNA sequencing analysis and filter for mutational burden assessment

The raw fastq files from the Illumina HiSeq platform were aligned to the human reference genome GRCh38 using BWA MEM version 0.7.10^32^. The aligned BAM files were used to call variants using Haplotype Caller from GATK version 4.4–46^33^. To obtain high quality variants we filtered the positions that had depths of coverage less than 20, minor-allele frequency less than 10% and a genotype quality (GQ, encoded as a phred quality such that the higher the GQ, the higher the likelihood of true positive) value less than 30. In order to exclude variants that occur in the normal population we filtered any positions which were reported in the dbSNP^34^ or present in the 1000 genome^35^ or ExAC^36^ database with more than 2% frequency. On top of these filters we used only non-synonymous mutations. The mutation burden was calculated as the number of mutations per megabase of sequenced region after applying all the above filters.

### Method for neoantigen detection

For neoantigen detection, the filtered variants were used to generate peptide sequences of different lengths (8–12 mers) using a custom script and HLA type of the individual was determined using the matching normal sample whenever available using Polysolver version v1.0^37^. In cases where there was no matching normal tissue, the matching primary tumor was used. The HLA type and peptide sequences from respective individuals were used to predict the binding affinity between the normal and the mutated peptide sequence using NetMHC^38^ which uses a machine learning algorithm to generate an affinity score. Only the mutations which had a 10 fold affinity over the normal peptide were called as neoantigen. This number was used to calculate the neoantigen burden.

### Immunohistochemistry (IHC)

IHC for CD3 was performed as we have done previously^12,13^. Blocks were sectioned at 5 microns. Deparaffinization and IHC staining were performed on-line. Staining for CD3 was performed on the Ventana Benchmark XT (Ventana Medical Systems, Tucson, Arizona). CD3, Mouse Monoclonal (Clone LN10, Leica, Buffalo, IL, #NCL-L-CD3-565) was diluted 1/250 and incubated for 15 minutes at 37 °C. OptiView DAB (Ventana Medical Systems, Tucson, Arizona) was used for detection. Normal tonsil was used as positive control and normal tonsil without primary antibody was used as a negative control. The number of CD3^+^ tumor-infiltrating lymphocytes were counted and averaged over three high-powered fields.

### Heterogeneity indices

Richness (z) was defined as the number of unique T cell clones based on nucleotide sequence unless otherwise noted. Since a section of tumor was used instead of the whole tumor, iChao1 and Efron-Thisted esitmators were used to estimate total T cell richness within a lesion. Various means have been proposed to estimate the total number of species, or in this case T cell clones, even if they are not detected during sampling, including the widely used nonparametric approach developed by Chao^39^. As the estimate proposed by Chao relies only on clones detected once or twice, iChao1 has been developed as an “improved” estimator of species richness and includes the clones that were detected three or four times in the calculation^40^. The improved estimate is defined as

1 $${\rm{iChao1}}={z}_{obs}+\,\frac{(n-1)}{n}\frac{{f}_{1}^{2}}{2{f}_{2}}+\frac{{f}_{3}}{4{f}_{4}}\,\times \,{\rm{\max }}({f}_{1}-\frac{{f}_{2}{f}_{3}}{2{f}_{4}},0)\,$$

where *f*_*i*_ represents the number of clones detected i times and n represents the sum of the sampled clonal frequencies (*X*_*i*_) such that

2 $${\rm{n}}=\sum _{i=1}^{z}{X}_{i}$$

The Efron-Thisted Estimator is described elsewhere^41^. In the context of our data, both iChao1 and the Efron-Thisted Estimator provide an estimate of the total number of unique T cell clones in each lesion, which is similar to the estimation of species richness in the original works.

We applied Pielou’s Evenness Index (J′) in order to understand whether T cell clones were equally distributed amongst specimens42. This is defined as

3 $$J^{\prime} =\frac{H^{\prime} }{{H}_{max}^{\text{'}}}$$

where ${H}^{\text{'}}$ represents the Shannon Diversity Index

4 $$(H^{\prime} =-\sum _{i=1}^{z}{p}_{i}\,\mathrm{ln}\,{p}_{i})$$

and ${H}_{max}^{\text{'}}$ is the richness (z)^43^. *J*′may range from 0 to 1, where 0 represents less variation in the abundance of clones and 1 represents great variation in clonal abundance. *p*_*i*_ represents the proportion of the *i*th species in the population. In the setting of this work, Simpson’s Diversity Index (λ) represents the probability that two T cells taken at random from a specimen represent the same clone. This index is defined as

5 $$\lambda =\sum _{i=1}^{z}{\pi }_{z}^{2}\,$$

where z is the richness or the number of unique T cell clones, and π is the proportional abundance (percent of total) of each clone^44^. Due to the richness of T cell clones that we observed in our specimens, and the influence many rare clones may have on Pielou’s Evenness and Simpson’s Diversity Indices, the proportional abundance of the ten most abundant clones relative to all clones in each specimen was also determined. Morisita’s index was used to compare overlap between samples and was defined as

6 $$M=\frac{2{\sum }_{i=1}^{z}{x}_{i}{y}_{i}}{({{\rm{\lambda }}}_{x}+{{\rm{\lambda }}}_{y})XY}$$

where *x*_*i*_ represents the number of times T cell clone *i* is represented in the total X from the lung primar*y*, *y*_*i*_ represents the number of times T cell clone *i* is represented in the total Y from the paired brain metastasis, and *λ*_*x*_ and *λ*_*y*_ represent Simpson’s Diversity Index λ for each paired lesion^45^.

The concordance of mutations between paired specimens was calculated with

7 $$(\frac{S}{S+\frac{(U1+U2)}{2}})\ast 100$$

such that S is the number of shared mutations, U1 is the number of unique mutations in the primary lesion and U2 is the number of unique mutations in the metastatic pair as has been done previously^46^.

### Statistical comparisons

Descriptive statistics were used to describe patient characteristics and to summarize results. The paired t test was used to compare tumor-infiltrating lymphocytes (TILs), heterogeneity indices and estimators, tumor mutation burden and predicted neoantigen load between paired primary and metastatic NSCLC lesions. Spearman’s rank correlation was used where noted and its significance determined with a two-tailed test. P values < 0.05 were considered significant. Prism 7 for Mac OS X (GraphPad Software, Inc.) was used for this test. The hive plot^47^ was generated with an online tool provided by the Wodak laboratory at The Hospital for Sick Children Toronto, Canada (http://www.wodaklab.org/hivegraph/ accessed on 29 August 2017). This project was approved by Mayo Clinic’s Institutional Review Board (#13-007990) and all experiments were performed in accordance with the relevant guidelines and regulations.

### Data sharing

These T cell receptor sequences and additional data on T cell clones will be listed by the DOI, manuscript title, and the name of the primary author through Adaptive Biotechnologies’ immuneACCESS Platform: https://clients.adaptivebiotech.com/immuneaccess.

## Electronic supplementary material

Supplementary Dataset 1
